# Asymptomatic intrahepatic gossypiboma incidentally discovered: A case report

**DOI:** 10.1016/j.radcr.2025.07.025

**Published:** 2025-08-08

**Authors:** Chaimae Abourak, Ihssane Laasri, Saleck Choumad, Siham Oukassem, Bilal Motassim billah, ttimade Nassar, Kaoutar Imrani

**Affiliations:** Central Radiology Department, Ibn Sina University Hospital, Faculty of Medicine and Pharmacy of Rabat, Mohammed V University, Rabat, Morocco

**Keywords:** Gossypiboma, Textiloma, Intrahepatic mass, MRI, CT, Surgical history

## Abstract

Gossypiboma, also known as textiloma, refers to a retained surgical sponge or textile material left in the body following a surgical procedure. Intrahepatic localization is extremely rare and can mimic various hepatic lesions, posing a diagnostic challenge, particularly in asymptomatic patients. We report the case of a 63-year-old woman with a history of cholecystectomy and hepatic cyst surgery, who was referred for routine follow-up after a spontaneously resolved episode of jaundice. Laboratory investigations were unremarkable. Magnetic resonance cholangiopancreatography (MRCP) revealed mild biliary dilation without obstruction and identified a well-defined heterogeneous lesion at the surgical margin. The lesion exhibited characteristic imaging features across multiple modalities, including fat content, peripheral enhancement, and internal calcifications. Based on these findings and the patient’s surgical history, a diagnosis of intrahepatic gossypiboma was established. As the patient remained asymptomatic, a multidisciplinary team opted for conservative management with clinical and radiological follow-up. This case emphasizes the importance of considering gossypiboma in the differential diagnosis of hepatic masses in patients with a history of abdominal surgery, and highlights the essential role of multimodal imaging in achieving an accurate diagnosis and guiding appropriate management.

## Introduction

Gossypiboma, also referred to as textiloma, is a rare but serious postoperative complication resulting from the inadvertent retention of a surgical sponge or textile material within the body, typically encapsulated by a foreign-body granulomatous reaction. Although its true incidence is likely underestimated due to medico-legal concerns, published reports suggest it may occur in approximately 1 in 1000 to 1 in 10,000 surgical procedures, most commonly following abdominal or pelvic surgeries. Intrahepatic localization is extremely uncommon and presents a diagnostic challenge, particularly in asymptomatic patients [1].

The clinical presentation of gossypiboma is highly variable. Some patients develop acute symptoms such as pain, fever, or signs of infection or obstruction shortly after surgery. However, many remain asymptomatic for years, with the retained foreign body discovered incidentally on imaging performed for unrelated reasons. Imaging modalities such as ultrasound, computed tomography (CT), and magnetic resonance imaging (MRI) are critical for diagnosis, as gossypibomas can mimic neoplastic, infectious, or cystic lesions depending on their composition and surrounding reaction [2].

Awareness of this condition is essential not only among surgeons but also among radiologists, to reduce avoidable morbidity and prevent unnecessary invasive procedures. Here, we report a rare case of an asymptomatic intrahepatic gossypiboma discovered incidentally in a patient with a prior history of hepatic cyst surgery, emphasizing the diagnostic role of multimodal imaging in such atypical postoperative findings.

## Case report

A 63-year-old woman, with a history of laparoscopic cholecystectomy in 2016 and hepatic cyst resection involving segments II and III in 2020, was referred to the gastroenterology (GIT) outpatient clinic for follow-up after a transient episode of cutaneous and scleral jaundice that had occurred approximately 1 year earlier. This episode lasted for about 4 days, resolved spontaneously without medical intervention, and was not accompanied by abdominal pain, fever, or digestive symptoms. No further episodes were reported, but given her history of hepatobiliary surgery, a structured clinical and paraclinical follow-up was initiated.

At the time of her first GIT consultation, the patient was asymptomatic. Physical examination revealed no abnormalities, with no palpable mass or organomegaly. Laboratory tests were requested to investigate the prior episode of jaundice and to evaluate overall liver function. The results were as follows:

Total bilirubin: 0.7 mg/dL (normal range: 0.2-1.2 mg/dL)

Direct bilirubin: 0.2 mg/dL (normal <0.3 mg/dL)

Aspartate aminotransferase (AST): 24 IU/L (normal <35 IU/L)

Alanine aminotransferase (ALT): 22 IU/L (normal <45 IU/L)

Alkaline phosphatase (ALP): 85 IU/L (normal range: 45–120 IU/L)

Gamma-glutamyl transferase (GGT): 34 IU/L (normal <38 IU/L)

Serum electrolytes, complete blood count, and C-reactive protein were all within normal limits.

**Fig. 1:**
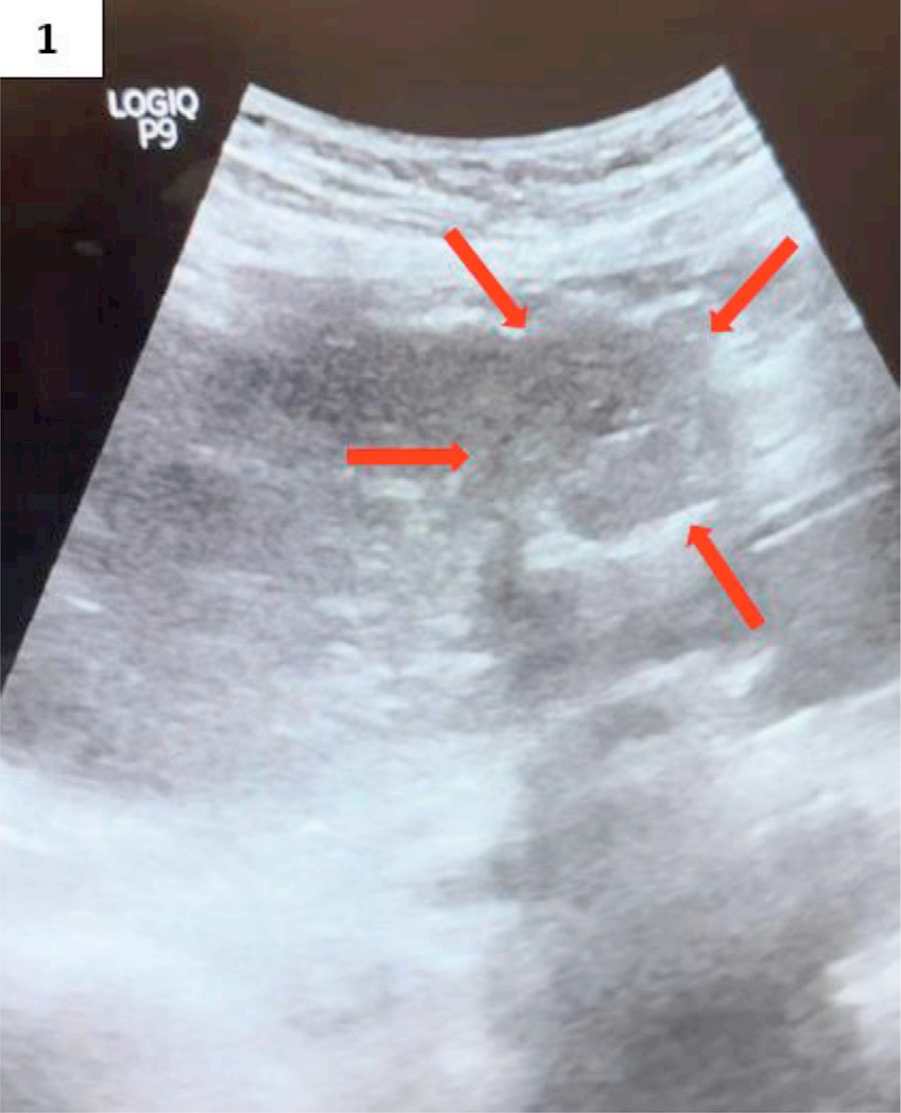
Abdominal ultrasound. Well-defined, oval-shaped mass with regular contours and heterogeneous hypoechoic echotexture, containing calcifications causing posterior acoustic shadowing (red arrows).

As part of the follow-up protocol, an abdominal ultrasound was performed 2 weeks later. It revealed mild dilation of the intrahepatic bile ducts and a rounded, heterogeneous, hypoechoic lesion measuring approximately 22 × 26 mm in segment IV, adjacent to the previous surgical site. The lesion contained punctate calcifications and demonstrated posterior acoustic shadowing, raising suspicion for a foreign body or atypical postoperative collection. Fig. 1.

Based on these findings, magnetic resonance cholangiopancreatography (MRCP) was indicated not as part of routine follow-up, but to further characterize the lesion and assess for possible biliary obstruction. MRCP was performed within 1 month of the ultrasound and revealed moderate dilation of the common bile duct and proximal intrahepatic bile ducts, without evidence of obstructive pathology. It also confirmed the presence of a well-defined, rounded lesion at the surgical margin. The lesion appeared hypointense on T2-weighted sequences, isointense on T1-weighted images, and contained fat signal with drop-out on in-phase/out-of-phase sequences. Mild peripheral enhancement was noted after gadolinium administration (Fig. 3).

**Fig. 2:**
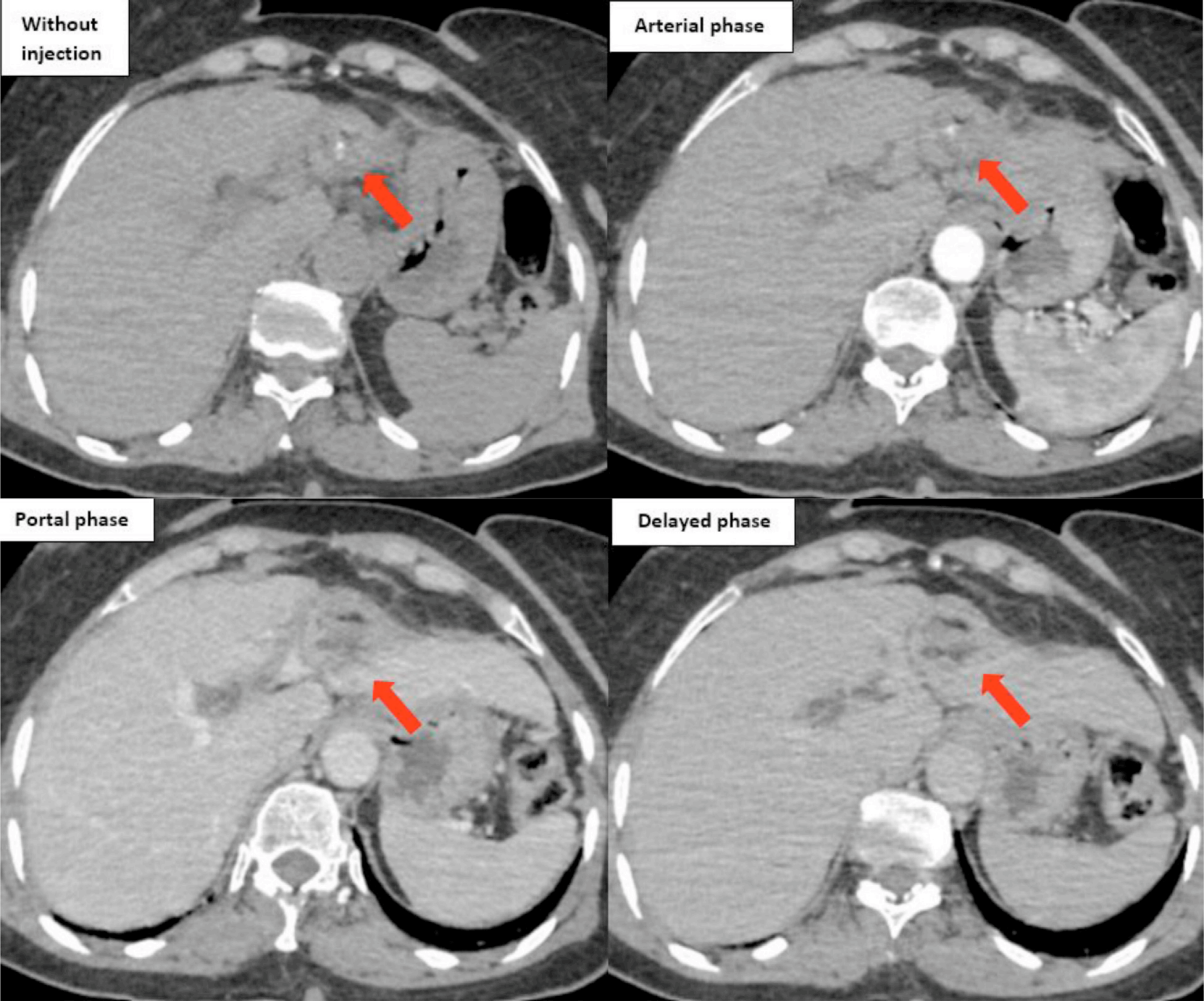
Contrast-enhanced abdominal CT scan (unenhanced and triphasic acquisition). Well-defined, rounded lesion located in hepatic segment IV, appearing hypodense, containing both fatty components and calcifications, with mild peripheral enhancement observed during the portal venous phase following intravenous iodinated contrast injection (red arrow).

To complete the assessment, a contrast-enhanced abdominal CT scan was performed 2 weeks later. It showed a hypodense lesion in segment IV, with internal areas of fat attenuation, coarse calcifications, and mild peripheral enhancement, consistent with a chronic foreign body reaction (Fig. 2).

**Fig. 3:**
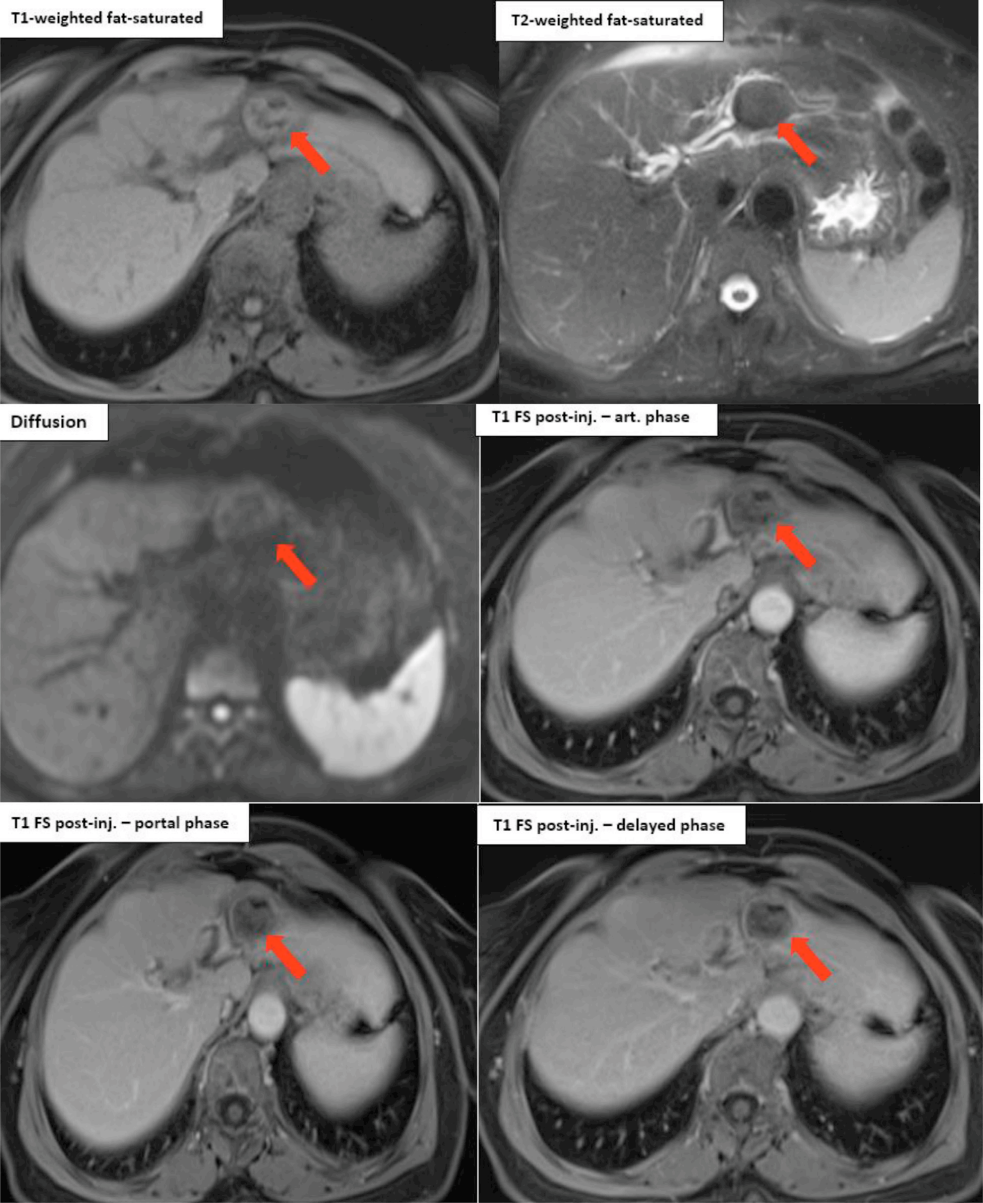
MRI of the liver. Post-segmentectomy changes of segments II and III, with identification of a well-defined, rounded lesion at the resection margin. The lesion appears hypointense on T2-weighted images, isointense on T1-weighted images, contains fatty areas showing signal drop on Dixon in-phase/out-of-phase sequences, and demonstrates moderate peripheral enhancement after contrast administration (red arrow).

The multimodal imaging characteristics, combined with the patient’s prior hepatic surgery and stable clinical status, led to the diagnosis of an intrahepatic gossypiboma. After multidisciplinary discussion involving radiologists, hepatologists, and surgeons, conservative management was recommended. A follow-up protocol involving regular clinical examinations, liver function tests, and imaging surveillance was implemented.

## Discussion

Gossypiboma, first described by Wilson in 1884, refers to the inadvertent retention of a surgical sponge or swab inside the body following an operation. Although rare, the actual incidence is likely underreported due to medico-legal implications, with published estimates ranging from 1 in 1000 to 1 in 5,000 surgeries. Retained foreign bodies account for up to 50% of surgical malpractice claims [3]. In a comprehensive review, Wan et al. identified 254 reported cases over a 45-year period, with the majority (56%) involving the abdominal cavity. However, cases involving the liver are extremely rare, and only 2 truly intrahepatic cases have been documented in the literature [4].

Surgical sponges, typically composed of cotton, provoke a foreign-body reaction rather than a specific biochemical response. The clinical presentation of gossypiboma varies widely, depending on its location, the body's immune response, and the time since surgery. The body may react through a sterile fibrous response—leading to encapsulation, adhesions, and granuloma formation—or through an exudative inflammatory process, which can cause abscess formation, fistulas, or even migration into hollow viscera. In rare cases, retained sponges have migrated into the gastrointestinal tract and passed spontaneously via the ileocecal valve [3].

Symptoms can manifest immediately postoperatively or remain absent for decades. Presentations range from nonspecific abdominal discomfort and palpable masses to fever or a pseudotumoral appearance. Intrahepatic gossypibomas have occasionally been associated with jaundice or biliary obstruction [4,5]. However, approximately one-third of patients remain asymptomatic, as was the case with our patient, in whom the retained sponge was discovered incidentally during imaging performed as part of routine postoperative surveillance.

Imaging is essential for diagnosis. Plain radiographs can be helpful if the sponge contains radio-opaque markers, although many retained sponges do not. Ultrasound may reveal a cystic or heterogeneous mass with characteristic strong posterior acoustic shadowing due to the presence of air, calcifications, or the sponge fibers themselves [3]. Computed tomography (CT) is the most effective modality for diagnosis and assessing associated complications. Key CT features include a well-circumscribed cystic mass with internal gas bubbles—the so-called “spongiform pattern”—and, as described by Lu et al., a ``calcified reticulate rind'' sign, where the lesion displays a thick, lace-like calcified rim due to gradual calcium deposition along the cotton fibers [1,6]. These imaging features, especially in a patient with prior abdominal surgery, strongly support the diagnosis.

Although magnetic resonance imaging (MRI) is less commonly used, it can provide additional information. Gossypibomas typically appear as well-defined lesions that are hypointense on T1-weighted and hyperintense on T2-weighted sequences, often surrounded by a low-signal-intensity capsule. However, radio-opaque markers used in sponges, such as barium sulfate, are not magnetic and produce little to no signal or artifact, limiting MRI sensitivity [7].

Our case shares features with previously reported intrahepatic gossypibomas, particularly in terms of imaging findings. However, the completely asymptomatic presentation and incidental discovery distinguish this case. These features underscore the critical role of imaging in detecting retained foreign bodies in postoperative patients, even in the absence of clinical symptoms.

The differential diagnosis for intra-abdominal gossypiboma includes postoperative hematoma, abscess, fecaloma, bowel obstruction, gastrointestinal stromal tumors (GIST), and recurrent or new malignancies. CT plays a central role in differentiation: abscesses usually present as fluid-density masses with enhancing walls and air-fluid levels, while gossypibomas often show the spongiform pattern with fixed gas bubbles and a thick capsule. Hematomas are more common early postoperatively and tend to resolve spontaneously. Fecalomas and bowel obstruction may mimic gossypiboma but typically lack the defining thick fibrous capsule [8].

Prevention is paramount. Current guidelines recommend strict sponge counting protocols—at the start and conclusion of surgery—conducted by at least 2 trained staff members, with audible confirmation. Some institutions advocate a third interim count. Additionally, maintaining a calm, well-organized surgical environment helps reduce cognitive errors and minimizes distractions, further lowering the risk of retention.

## Conclusion

Gossypiboma remains a rare but significant postoperative complication that can be difficult to diagnose, particularly in asymptomatic patients. Delayed detection may result in serious complications, including infection, obstruction, or fistula formation. Recognizing its characteristic imaging features—especially in patients with a history of prior surgery—is essential for prompt and accurate diagnosis. Since clinical suspicion is often low, radiologic evaluation plays a critical role in identifying retained surgical materials and guiding appropriate management.

## Patient consent

Written informed consent for the publication of this case report was obtained from the patient.
